# Potential ‘anti-cancer’ effects of esketamine on proliferation, apoptosis, migration and invasion in esophageal squamous carcinoma cells

**DOI:** 10.1186/s40001-023-01511-x

**Published:** 2023-11-15

**Authors:** Chao Li, Jingpu Shi, Sisi Wei, Huiqun Jia

**Affiliations:** 1 Department of Anesthesiology, The Fourth Hospital of Hebei Medical University, 12th Health Road, Shijiazhuang, Hebei 050011 People’s Republic of China; 2 Scientific Research Center, The Fourth Hospital of Hebei Medical University, 12th Health Road, Shijiazhuang, Hebei 050011 People’s Republic of China

**Keywords:** Esketamine, Esophageal squamous cell carcinoma, Proliferation, Apoptosis, Proteomics

## Abstract

**Background:**

Esketamine, an *N*-methyl-D-aspartate receptor antagonist, is commonly used for anesthesia and analgesia clinically. It was reported to negatively regulate cell proliferation, metastasis and apoptosis in cancer cells, including lung cancer and pancreatic cancer. However, its impact on esophageal squamous cell carcinoma (ESCC) malignance and underlying mechanism remain elusive. This study was aimed to investigate the antitumor effects of esketamine on ESCC in vitro.

**Methods:**

ESCC cell lines (KYSE-30 and KYSE-150) were cultured and treated with different concentrations (0.1, 0.2, 0.4, 0.8, 1, 2 mM) of esketamine. Their proliferation, apoptosis, migration and invasion were assessed with various assays. Furthermore, mass spectrometry-based proteomic analysis and GO/KEGG enrichment analysis were applied to characterize the differentially expressed proteins (DEPs) with or without esketamine treatment. Some key proteins identified from proteomic analysis were further validated with Western blotting and bioinformatics analysis.

**Results:**

Esketamine significantly inhibited the proliferation, migration, invasion and promoted apoptosis of the both types of cell lines in a dose- and time-dependent manner. A total of 321 common DEPs, including 97 upregulated and 224 downregulated proteins, were found with HPLC–MS analyses. GO/KEGG enrichment analysis suggested that esketamine affected cell population proliferation, GTPase activity and Apelin signaling pathway. The ERCC6L, AHR and KIF2C protein expression was significantly downregulated in these ESCC cells treated with esketamine compared to the controls and their changes were associated with the suppressive effects of esketamine on ESCC through bioinformatics analysis.

**Conclusions:**

Our work demonstrated that esketamine has potential anti-ESCC properties in vitro but subjected to further in vivo and clinical study.

**Supplementary Information:**

The online version contains supplementary material available at 10.1186/s40001-023-01511-x.

## Background

Esophageal carcinoma is the 8th most common cancer and the 6th leading cause of cancer-related deaths worldwide [1]. Esophageal squamous cell carcinoma (ESCC) accounts up to 90% of all esophageal cancer cases globally and has a 5-year survival rate less than 20% [2]. A large number of ESCC survivors require comprehensive analgesic management to relives the devastating perioperative and cancer-related pain. Accumulating evidence demonstrated that commonly used analgesics may have potential effects on stress, immune system and cancer recurrence [3, 4], while general anesthetics may act to make miRNAs modification changing cancer cell biology [5]. In addition, opioids, such as morphine commonly used for severe pain, was reported to result in cancer proliferation, tumor microenvironment changes and chemoresistance [6–8]. Therefore, exploring safe and effective analgesics with potential anti-cancer properties for ESCC using during and/or after cancer surgery would be benefit to cancer patients.

Ketamine is a commonly used *N*-methyl-D-aspartate (NMDA) receptor antagonist clinically as an anesthetic, analgesic or sedative agent [9]. Despite its undesirable side effects, including dissociative effects and abuse potential, ketamine is currently favored by clinicians due to its fast-onset, short half-life, minimal suppressive effects on respiration and pronounced analgesic properties [10]. Recently, its new derivative esketamine with stronger NMDA receptor-binding affinity, potent analgesic and fewer side-effects than ketamine was introduced into clinical practice. In addition to their analgesic effect [11], ketamine and esketamine have recently been reported to have potential antidepressant [12] and antitumor properties [13, 14] but underlying mechanisms remain unknown.

NMDA receptors were reported to be expressed in various cancers and associated with cancer initiation, disease progression and poor prognosis [15]. Furthermore, NMDA receptor antagonists, e.g., MK-801, decreased cancer cell viability and suppressed tumor cell growth [16]. The previous studies also showed that NMDA receptor antagonists such as ketamine and sketamine had anti-proliferative and anti-invasive effects in some cancers, including pancreatic cancer, colorectal cancer, ovarian cancer and neuroglioma [14, 16–18]. In this study, we aimed to investigate the potential suppressive effects of esketamine at different concentrations on proliferation, apoptosis, migration and invasion of two ESCC cell lines (KYSE-30 and KYSE-150) through proteomics, bioinformatics and western blotting analyses.

## Methods

### Cell culture and reagents

Human esophageal squamous cell carcinoma (ESCC) is very common clinically counted up to 90% esophageal cancer cases [2]. Therefore, two cell lines (KYSE-30 and KYSE-150) its phenotype, purchased from Procell Life Science&Technology Co., Ltd. (Wuhan, Hubei, China) and stored in the Dr. Baoen Shan’s laboratory from the Research Center of the Fourth Hospital of Hebei Medical University (Shijiazhuang, China) [19], were used in the present study. They were cultured with RPMI 1640 medium supplemented with 10% bovine serum. The cells were grown in monolayer at 37 °C, 5% CO_2_ balanced with air, and 60% humidity. Esketamine hydrochloride was purchased from Jiangsu Hengrui Pharmaceutical Co., Ltd (China). It was dissolved in normal saline, with the pH adjusted to 7.4, and kept at − 20 °C. When reaching 90% confluence, the cultured ESCC cells were treated with esketamine at the concentrations of 0.1, 0.2, 0.4, 0.8, 1 or 2 mM for various duration up to 72 h. Saline-treated cells were served as the control.

### Cell viability and proliferation assay

Cell viability was measured using 3-(4,5-dimethylthiazol-zyl)-5-(3-carboxymethoxyphenyl)-2-(4-sulfophenyl)-2H-tetrazoliuzolium, inner salt (MTS) assay described as previous research [20]. Briefly, KYSE-30 and KYSE-150 cells were seeded in 96-well plates (approximately 5 × 10^3^ cells/well) and then treated with the doses of esketamine (0, 0.4, 0.8, 1, 2 mM) up to 72 h [17, 21]. Those doses and the duration did not completely “kill” rather than injured cancer cells and hence the underlying mechanisms can be investigated. Next, 15 μL of MTS solution was added to each well followed by incubation for 2 h at 37 °C in the dark. Absorbance at 492 nm was measured using a microplate reader (Thermo Fisher Scientific Inc., MA, United States). The inhibition rates of ESCC was calculated as follows:

$$[{1} - \left( {{\text{OD esketamine treated}} - {\text{OD blank}}} \right)/\left( {{\text{OD control}} - {\text{OD blank}}} \right)]\, \times \,{1}00\% .$$

For colony formation assay, cells were seeded on 6-well plate at a seeding density of 1000 cells per well. After 2 weeks of incubation, visible colonies were fixed with 4% paraformaldehyde, stained with 1% crystal violet solution and then assessed.

### Apoptosis assay

The effect of esketamine on ESCC cell apoptosis was determined by flow cytometric analysis. Briefly, 5 × 10^5^ cells were seeded and washed with phosphate-buffered saline (PBS). Then, cells were resuspended in 500 µl of binding buffer and incubated with 5 µl annexin V (FITC) and 5 µl propidium iodide (PI) for 5 min at room temperature. Finally, cells were analyzed with flow cytometry (FACSCalibur; BD Biosciences).

### Wound healing assay

Cells were seeded into 6-well plates at a density of 5 × 10^5^ cells/well and cultured in serum-free medium. When cells grown to approximately 80% confluence, cell monolayers were scratched with a 200 μl pipette tip. The medium was then replaced with fresh medium containing 0, 0.05, 0.1, or 0.2 mM esketamine. At 0 h, 24 h and 48 h following scratching, each scratch was measured with an inverted microscope from three independent visual fields (40×).

### Transwell invasion assay

A total of 1 × 10^5^ cells with 0.2 ml serum-free RPMI 1640 medium were plated in the upper chamber (Boyden chamber; pore size of 8 μm; coated with 200 μg/ml Matrigel) (Beyotime Biotechnology), while the lower chamber was filled with 0.6 ml RPMI 1640 medium containing 10% fetal bovine as a chemoattractant. After 18 h incubation, non-invading cells on the upper surface of the upper chamber were removed, and the membrane-penetrating cells were stained in 0.5% crystal violet for 15 min. The number of stained cells that passed through the transwell membrane was counted from three independent visual fields (100×).

### Mass spectrometry-based proteomic analysis

To detect the proteins changes and underlying mechanism for the antitumor effect of esketamine, we performed liquid chromatography–mass spectrometry/mass spectrometry analysis as previously described [22]. Briefly, after incubated with 0, 1, 2 mM esketamine for 24 h, KYSE30 cellular proteins were extracted by SDT lysis buffer. The protein concentration was measured with the BCA protein assay. Then, we performed a filter-aided sample preparation (FASP) procedure to digest protein. After digestion, eluted peptides were further purified and extracted using homemade C18 tips (Empore) in 80% ACN and 2% TFA. Peptide quantification was performed using a BCA peptide quantification kit (Thermo). For proteomics analysis, 100 μg peptide from each sample was loaded onto the HPLC Easy nLC1200 system with a Q Exactive HF mass spectrometer (Thermo Fisher Scientific) based on the manufacturer’s instructions. Raw MS data were processed and analyzed in Proteome Discoverer (version 2.2, Thermo-Fischer Scientific) based on the label-free quantification method. All resulting MS data were uploaded and searched against UniProt Human database.

### Bioinformatic analysis of differentially expressed proteins

We first carried out differential protein expression analysis between the different doses of the esketamine groups with the control group to determine differentially expressed proteins (DEPs). The cutoff criteria for DEPs are |log_2_(FoldChange)|> 0.58 and *P* value < 0.05. After identification of DEPs, we used online web tool (https://cloud.oebiotech.com/task/) to perform their functional annotations, including the gene ontologies (GO) enrichment and Kyoto Encyclopedia of Genes and Genomes (KEGG) pathways enrichment. Top 20 of GO/KEGG enriched pathway were listed in figures, and numbers and color intensity indicated enrichment score. Then, we draw a Venn diagram of DEPs via xiantaoxueshu online tool (www.xiantao.love/).

The mRNA expression of these proteins in ESCA patients were extracted from GEPIA database (http://gepia.cancer-pku.cn/) [23]. Then, we constructed a network of the 5 selected proteins based on the STING interactome database (version 11.5) with medium confidence score 0.4 [24].

### Western blotting analysis

KYSE-30 and KYSE-150cells were treated with esketamine (0, 1, 2 mM) for 48 h. Then, the harvested cells were washed with PBS and lysed in ice-cold RIPA lysis buffer containing protease inhibitors to obtain the total protein. The cell lysates were cleared by centrifugation 4 °C and protein concentration was determined by BCA assay. Protein samples (40 μg) were separated on 10% SDS–PAGE and then transferred onto PVDF membrane (Millipore). After blocking the membranes with 5% BSA for 2 h at room temperature, they were incubated overnight at 4 °C with primary antibodies against ERCC6L (1:1000), AHR (1:1000), KIF2C (1:1000), KNTC1 (1:500), BCAT1 (1:1000) or GAPDH (1:2000). The HRP-labeled secondary antibody was anti-rabbit IgG and the protein bands were visualized in an Odyssey Infrared Imaging System (LI-COR). Each protein expression level was normalized to those of GAPDH. Antibodies against ERCC6L, KIF2C, BCAT1, AHR and GAPDH were purchased from Proteintech. Antibodies against KNTC1 was obtained from Abcam.

### Correlation analysis between ERCC6L, AHR and KIF2C with diagnostic performance, immune cell infiltration and clinicopathological characteristics of ESCA patients from TCGA database

The TCGA–ESCA mRNA expression profiles and corresponding clinical data were downloaded from the TCGA database (https://portal.gdc.cancer.gov). We then performed diagnostic ROC curve analysis using pROC” (v1.17.0.1) to evaluate the predictive value of these protein-coding mRNA expression levels in ESCC patients. We also compared these protein-coding mRNA difference between the groups divided by clinicopathological parameters, including clinical stages, age, race and body mass index. To evaluate the relationship of these protein-coding mRNA with tumor infiltration status of ESCA, we used ssGSEA algorithm in the “GSVA” (v1.34.0) R package [25, 26]. Then, the correlation coefficient and significance were determined with Spearman’s coefficient analysis.

### Statistical analysis

All data were expressed as the mean ± SEM, and one-way ANOVA followed by Tukey’s multiple comparisons test was done with GraphPad Prism 9.0 (GraphPad Software, USA). Differences with *P* values less than 0.05 were considered to be statistically significant. *P < 0.05; ** P < 0.01; *** P < 0.001.

## Results

### Esketamine inhibited the viability and proliferation of ESCC cell in vitro

**Fig. 1:**
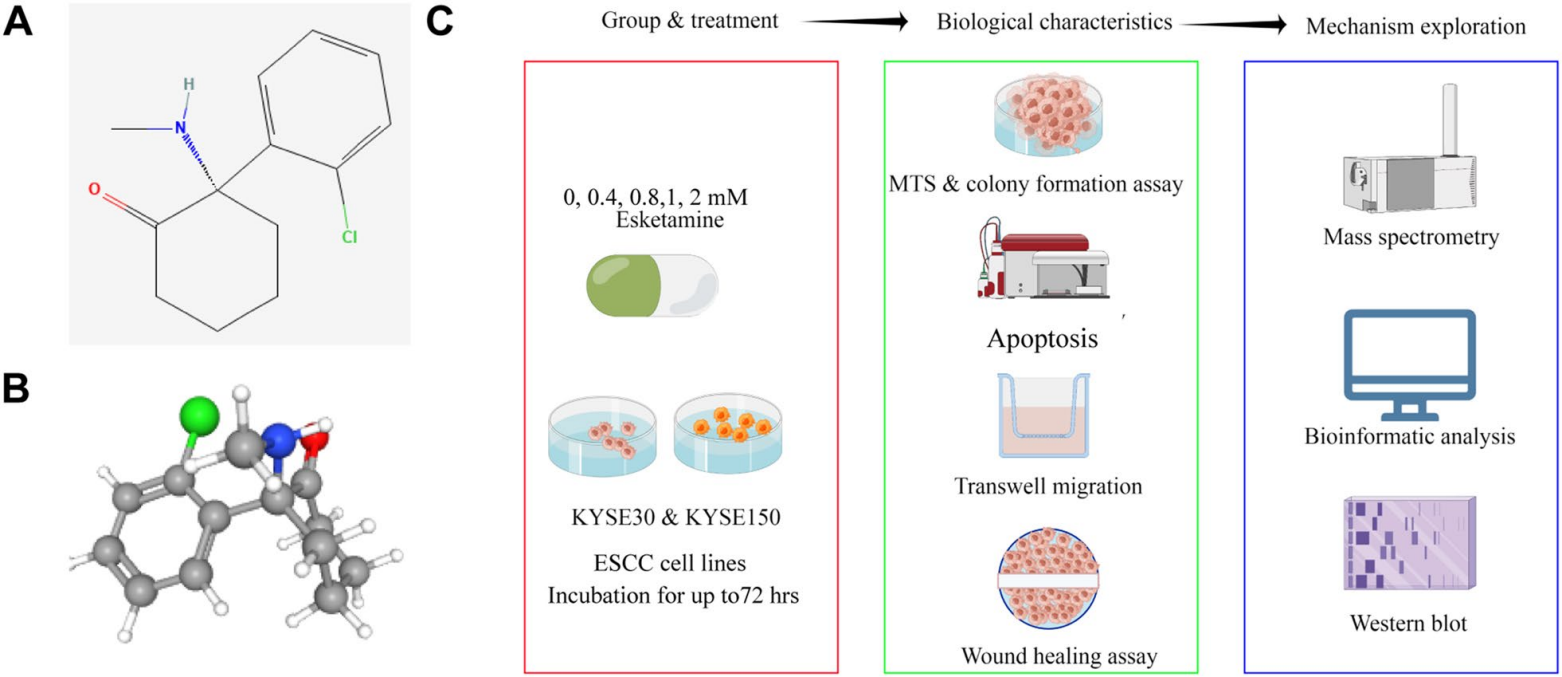
Molecular structure and 3D molecular structure of esketamine from PubChem database (**A**, **B**). Overview of the experimental workflow (**C**)

**Fig. 2:**
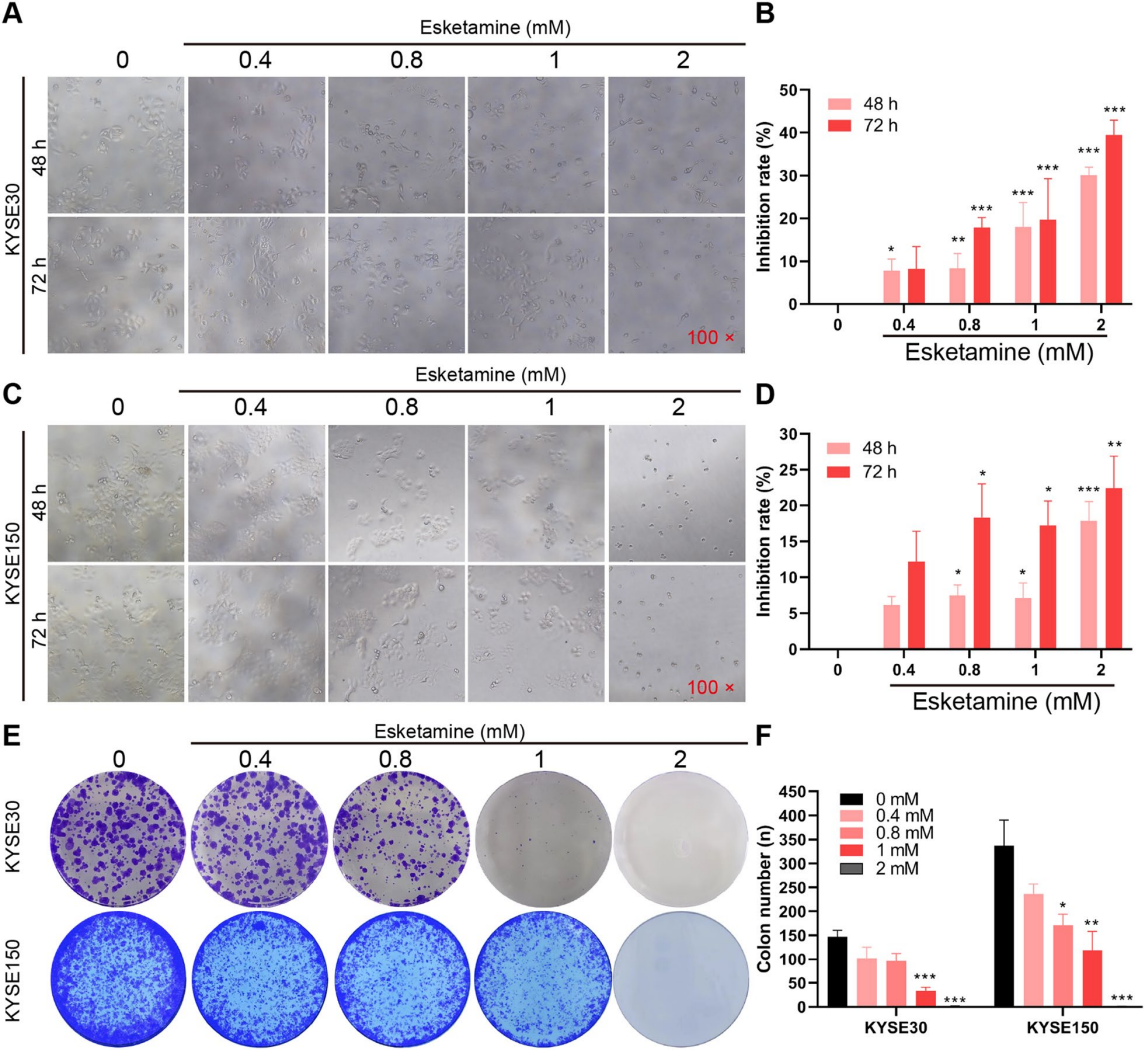
Esketamine suppressed the esophageal squamous carcinoma cells (ESCC) growth. ESCC KYSE-30 and KYSE-150 cells were exposed to concentration gradient of esketamine for 48 h or 72 h, and then cell proliferation was assessed with the MTS assay (**A**–**D**). Colony forming assays were performed after incubation with indicated concentrations of esketamine for 24 h (**E, F**). Data are presented as mean ± SEM; *P < 0.05, **P < 0.01, ***P < 0.001 vs the control group

The molecular structure and 3D molecular structure of esketamine were obtained from PubChem database and shown in Fig. 1A, B. The overall experimental procedures are shown in Fig. 1C. To assess the anticancer potential of esketamine on ESCC, we first investigated the effects of esketamine on the inhibition rate of ESCC cells, which were significantly upregulated after esketamine exposure for 48 h and 72 h (Fig. 2A–D). Moreover, ESCC cell growth inhibition increased with increasing concentration of esketamine. The inhibition rate of KYSE-30 and KYSE-150 tumor growth by 2 mM esketamine at 72 h was 39.50% and 22.44%, respectively. KYSE-30 was more prone to the inhibition by esketamine and the similar patten changes was found in the colony formation assay. As shown in Fig. 2E, F, the colonies number in both cell lines were concentration-dependently decreased after esketamine exposure.

### Esketamine promoted ESCC cell apoptosis

**Fig. 3:**
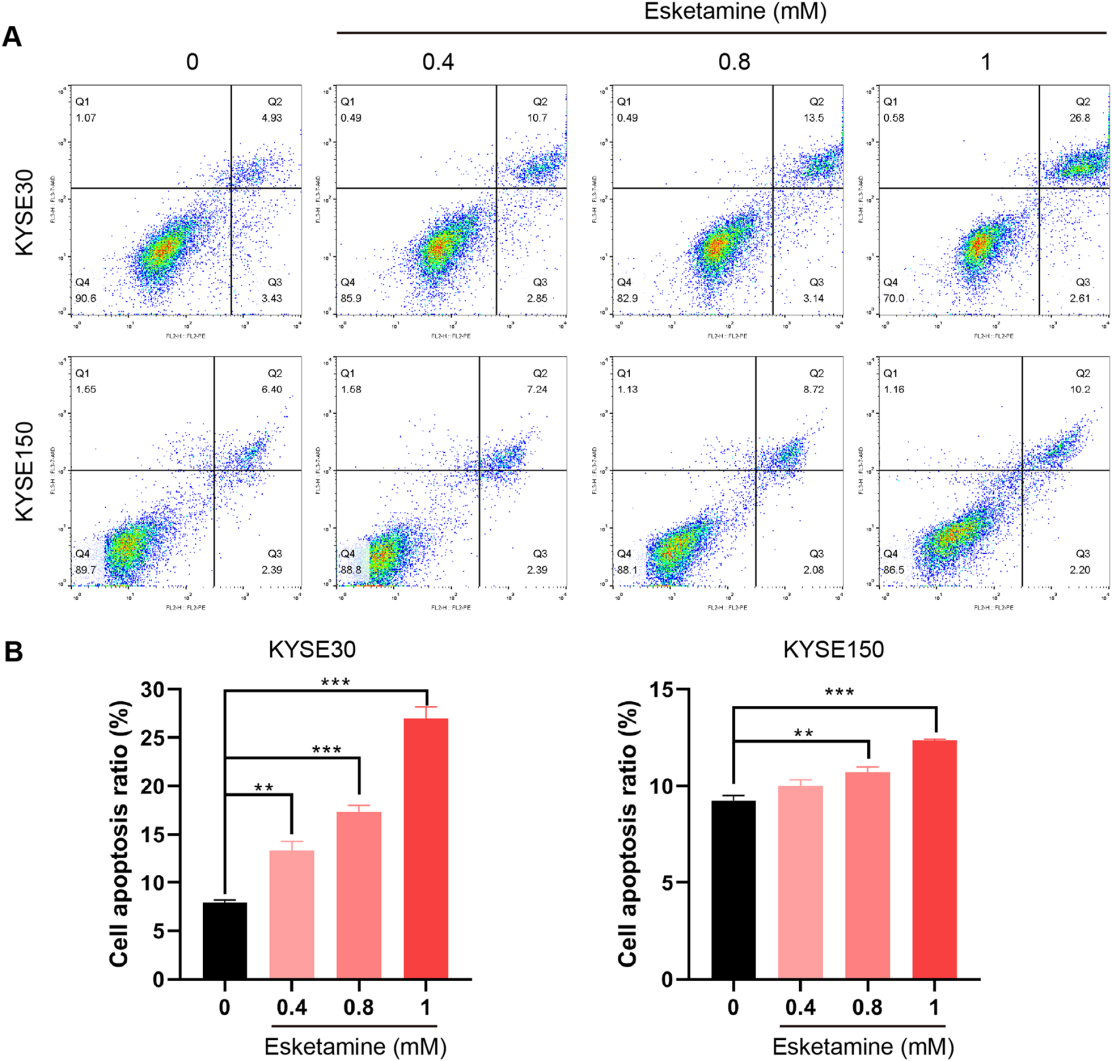
Esketamine induced the cell apoptosis changes in esophageal squamous carcinoma cells (ESCC). The effect of esketamine on apoptotic death of KYSE-30 cells and KYSE-30 cells were measured by flow cytometry (**A**). Chart (**B**) showed the statistical analysis of apoptosis ratio. Data are presented as mean ± SEM; **P < 0.01, ***P < 0.001 vs the control group

To determine whether the reduction in cell viability and proliferation was associated with apoptosis, we performed annexin V/PI staining assay. Consistent with previous reports [21], esketamine significantly decreased cell viability and proliferation with significantly increasing fractions of apoptotic cells (sub G1 phase) compared with the control group (Fig. 3A–D). The apoptosis rate of KSYE-30 and KYSE-150 by 1 mM esketamine was 26.96% and 12.37%, respectively.

### Esketamine suppressed the migration and invasion ability of ESCC cells

**Fig. 4:**
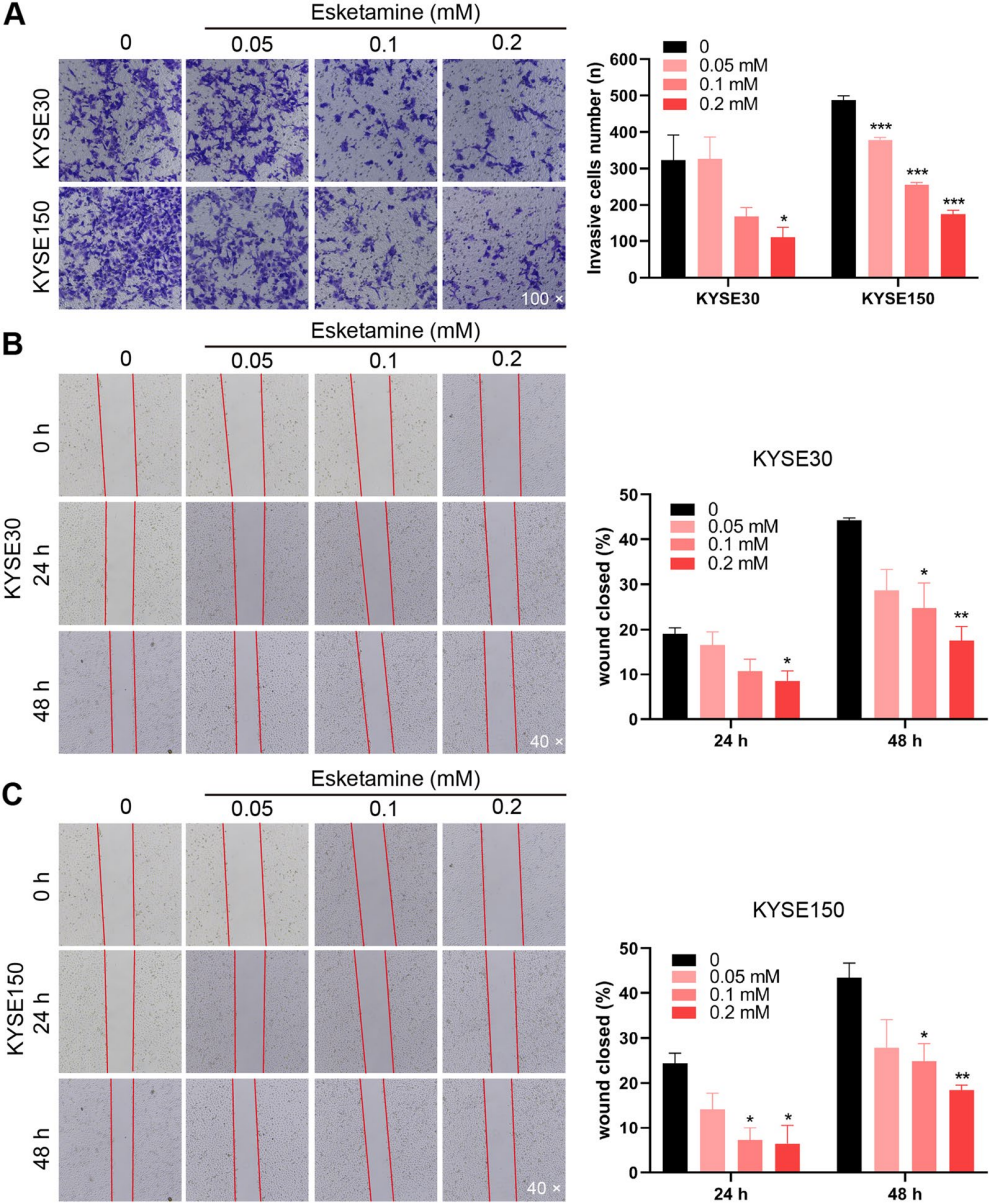
Esketamine treatment reduced the migration and invasion capabilities of esophageal squamous carcinoma cells (ESCC). Transwell invasion assay was implemented to examine the invasive ability of the KYSE-30 cells and KYSE-150 cells (**A**). Wound healing assays were conducted to detect the migratory of KYSE-30 cells (**B**) and KYSE-30cells (**C**). The migratory ratio was calculated by dividing the wound area by total area. Data are presented as mean ± SEM; **P < 0.01, ***P < 0.001 *vs* the control group

In transwell cell invasion assay, the number of cells invading the Matrigel coated membrane in the esketamine groups was significantly lower than that of the control group, and the cell invasion ability was notably weakened (Fig. 4A). Similar findings were found in the wound-healing assay (Fig. 4B, C).

### The proteomic changes in ESCC cells induced by esketamine treatment

**Fig. 5:**
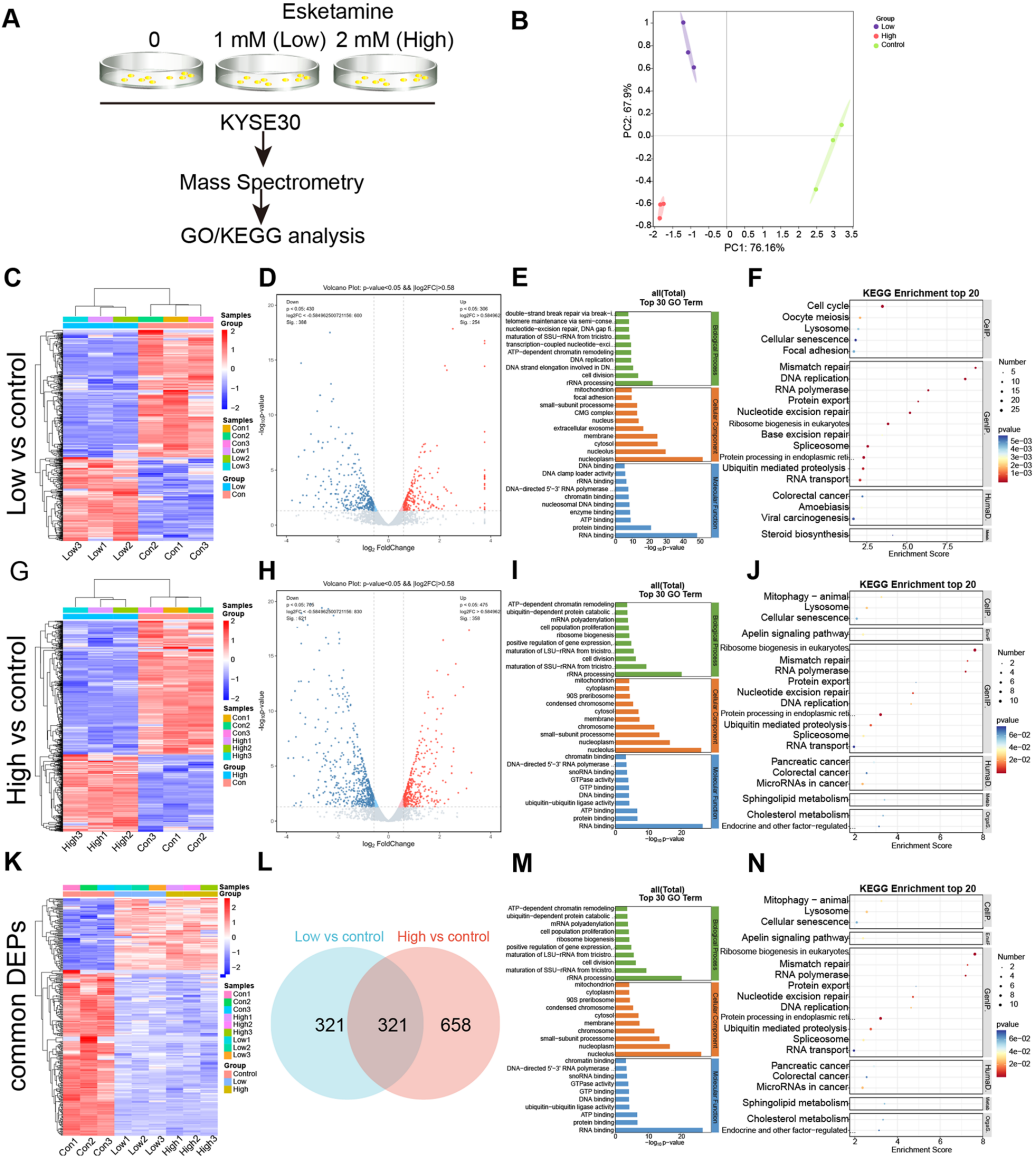
Proteomic changes of esophageal squamous carcinoma cells (ESCC) KSYE30 cells treated with esketamine. **A** Schematic workflow for the analysis of differential expressed proteins (DEPs) via mass spectrometry. **B** PCA analysis checked the grouping situation of nine samples from three different groups. The heatmap (**C**), volcano map (**D**), GO analysis (**E**) and KEGG analysis (**F**) for DEPs between KYSE30 treated with 0 or 1 Mm esketamine. The heatmap (**G**), volcano map (**H**), GO analysis (**I**) and KEGG analysis (**J**) for DEPs between KYSE30 treated with 0 or 2 mM esketamine. **K** Heat map for DEPs in differential groups of KSYE30 cells treated with increasing dose of esketamine (0, 1, 2 mM). Venn diagram (**L**) showed the common DEPs between 0 vs 1 mM ketamine group and 0 vs 2 mM ketamine group. GO analysis (**M**) and KEGG analysis (**N**) for these common DEPs

We performed TMT-based quantitative proteomics to examine the protein changes of ESCC KYSE30 cells under 0, 1 or 2 mM esketamine (Fig. 5A). Principal component analysis (PCA) of protein expression levels showed that the 9 samples segregated by experimental conditions, indicating that the samples were profiled without significant measurable bias or batch effect (Fig. 5B). We further compared these two groups (1 vs 0 mM esketamine) and identified 642 significant DEPs (Fig. 5C, D). Then, we further explored protein-wide expression changes assessed with the GO/KEGG enrichment analysis. The GO enrichment analysis, including biological processes, cellular component and molecular functions, found that the GO terms were enriched in rRNA processing, nucleolus and RNA binding (Fig. 5E). The KEGG pathway enrichment analysis showed a high abundance of endocytosis, nucleotide excision repair, MicroRNA in cancer and estrogen signaling pathway (Fig. 5F).

Similarly, we identified 979 significant DEPs in the KSYE30 cells incubated with 2 vs 0 mM esketamine (Fig. 5G, H). The GO enrichment analysis showed that the GO terms were enriched in cell division, rRNA processing and RNA binding, Fig. 5I). The KEGG pathway enrichment analysis also showed a high abundance of cell cycle, DNA replication and viral carcinogenesis (Fig. 5J).

We further mapped the intersection and determined 321 common DEPs in the three groups (KYSE30 cells under 0, 1, 2 mM esketamine). The GO enrichment analysis showed that the GO terms were enriched in cell division, rRNA processing, nucleoplasm, nucleolus, protein binding, RNA binding. The KEGG pathway enrichment analysis showed a high abundance of mitophagy, cellular senescence, apelin signaling pathway and DNA replication. Furthermore, 224 downregulated proteins following esketamine treatment of these 321 common DEPs were then analysed through the GEPIA database (http://gepia.cancer-pku.cn/detail.php) and found 32 commonly downregulated DEPs (Additional file 1: Table S1) which were also highly expressed in ESCC tissue from the GEPIA database. Of those, ERCC6L, AHR, KIF2C, KNTC1 and BCAT1 were reported to play significant roles in multiple cancers [27–32] and then chosen for further verification with Western blotting and bioinformatics analysis.

### Further analysis of key proteins and western blot verification

**Fig. 6:**
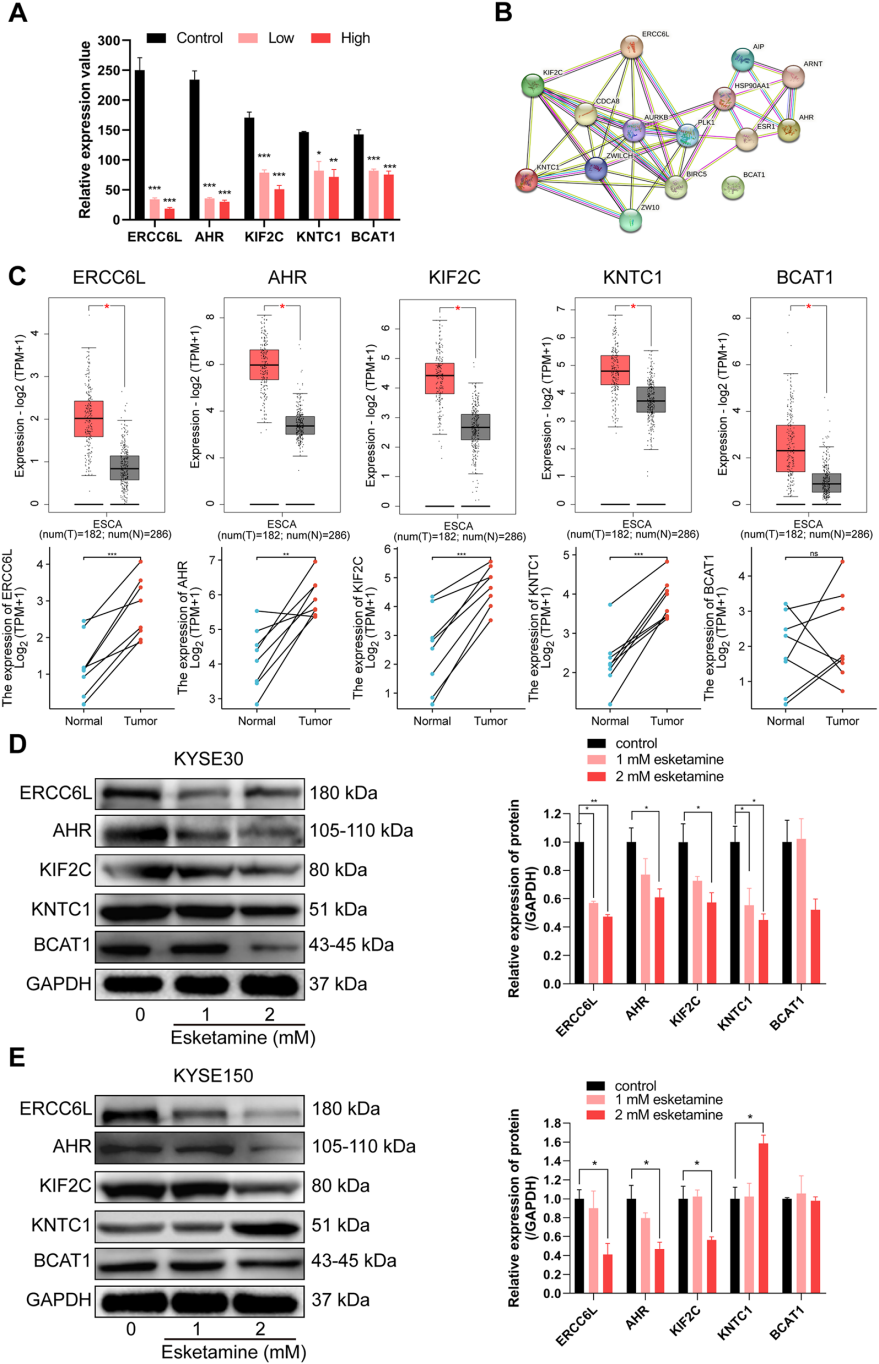
Further analysis of key proteins and western blot verification. **A** Histogram showed actual expression of ERCC6L, AHR, KIF2C, KNTC1 and BCAT1 in our proteomic data of KYSE30 cell after esketamine treatment. **B** Protein–protein interaction network for these key proteins from STRING database. **C** Expression levels of ERCC6L, AHR, KIF2C, KNTC1 and BCAT1 in the ESCA tissues and normal tissues from TCGA–ESCA data sets. Western blot analysis of ERCC6L, AHR, KIF2C, KNTC1 and BCAT1 in KYSE30 (**D**) and KYSE150 (**E**) under esketamine treatment. Data are presented as mean ± SEM; *P < 0.05, **P < 0.01, ***P < 0.001 *vs* the control group

Our proteomics results showed that these 5 proteins were significantly down-regulated in the KYSE30 cells treated with esketamine compared to these in the controls (Fig. 6A). These 5 were coherent “interaction” shown by the database of STRING (Fig. 6B). In addition, we further performed bioinformatic analysis from TCGA database and found that the expressions of ERCC6L, AHR, KIF2C, KNTC1 and BCAT1 were significantly higher in the ESCA tissues compared to the normal tissues (Fig. 6C). The protein expression of ERCC6L, AHR and KIF2C but not KNTC1 and BCAT1were significantly down-regulated after esketamine treatment. (*P < 0.05; ** P < 0.01, Fig. 6D, E).

### Correlation analysis between ERCC6L, AHR and KIF2C with diagnostic performance, immune cell infiltration and clinicopathological characteristics ESCA patients

**Fig. 7:**
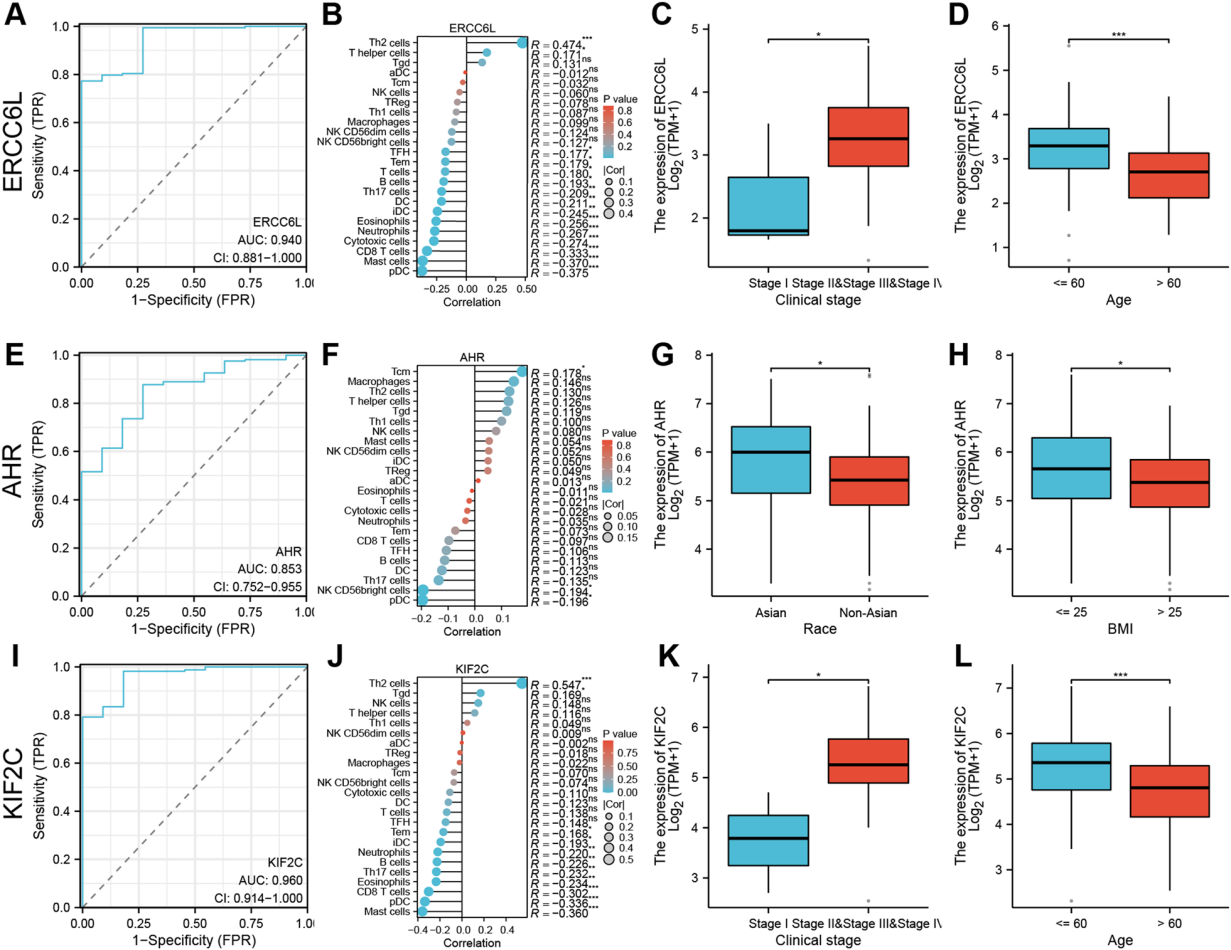
Correlation analysis between ERCC6L, AHR and KIF2C with diagnostic performance, immune cell infiltration and clinicopathological characteristics ESCA patients. Diagnostic ROC curves to discriminate ESCA tissue from normal tissues based on the expression levels of ERCC6L (**A**), AHR (**E**) and KIF2C (**I**). Spearman’s correlation analysis between immune cell infiltration levels and expression levels of ERCC6L (**B**), AHR (**F**) and KIF2C (**J**) in the ESCA tissues. The correlation analysis between ERCC6L and KIF2C expression levels and clinical stages and age (**C**, **D**, **K**, **L**). The correlation analysis between AHR expression levels and races and BMI (**G**, **H**)

We performed a ROC curve analysis to access the diagnostic value of ERCC6L, AHR and KIF2C. The expression levels of ERCC6L, AHR and KIF2C accurately distinguished ESCA tissues from normal tissue (respective AUC: 0.940, 0.853 and 0.960; Fig. 7A, E, I).

We then performed ssGSEA and Spearman analysis to determine the infiltration status of 24 immune cell types with the expression levels of ERCC6L, AHR and KIF2C in the ESCA tissues. ERCC6L expression levels showed a negative association with pDC (*R* =  − 0.375, *P* < 0.001), mast cells (*R* =  − 0.370, *P* < 0.001) and CD8 T cells (*R* =  − 0.333, *P* < 0.001), and positive association with aDCs (*R* = 0.474, *P* < 0.001) (Fig. 7B). AHR expression levels showed a negative association with pDC (*R* =  − 0.196, *P* < 0.05) and NK CD56bright cells (*R* =  − 0.194,* P* < 0.05), and positive association with Tcm (*R* = 0.178, *P* < 0.05) (Fig. 7F). KIF2C expression levels showed a negative association with mast cells (*R* =  − 0.360, *P* < 0.001), pDC (*R* =  − 0.336, *P* < 0.001) and CD8 T cells (*R* =  − 0.302, *P* < 0.001), and positive association with Th2 cells (*R* = 0.547, *P* < 0.001) (Fig. 7J).

Furthermore, we download the TCGA–ESCA data set to explore the association between the expression levels of ERCC6L, AHR and KIF2C and ESCA with patients’ multiple clinicopathological characteristics, including clinical stages, age, race, BMI and so on. Results showed that ERCC6L and KIF2C expression levels showed a significant correlation with the clinical stages (Fig. 7C, K) and age (Fig. 7D, L) of the ESCA patients. AHR expression levels showed a significant correlation with the race (Fig. 7G) and body mass index (Fig. 7H) of the ESCA patients.

## Discussion

Our data indicated that esketamine inhibited cell proliferation in a dose-dependent manner, which was accompanied by a significant increase in the number of apoptotic cells. Therefore, we deduced that the decreased cell proliferation of ESCC cells may be a partial consequence of increased cell apoptosis. Moreover, we also found that esketamine treatment significantly inhibited the migration and invasion ability at concentrations lower than that required to inhibit the proliferation of ESCA cells. Consequently, these findings supported that esketamine may have potential to possess anti-cancer properties for ESCC.

Esketamine, the s-enantiomer of ketamine, has superior analgesic effect and less psychotomimetic side effects than ketamine [33]. In addition to the high potential in relieving cancer pain [34] and depression [35], esketamine and ketamine also showed anti-cancer potential in numerous cancers, including pancreatic cancer, ovarian cancer and breast cancer [17, 18, 21] and its effect on ESCC was extended in our study.

High-throughput proteomic analysis can effectively detect protein changes to gain insight into pathophysiological mechanisms of cancers. Li and his colleagues performed integrative proteogenomic analysis of multiple tissues from 154 ESCC patients to elucidate cancer-driving waves and other crucial characterization of early esophageal cancer [36]. In the present study, we performed a proteomics analysis to identify proteins that were associated with cancer progression to probe the possible mechanism for the anti-cancer effects of esketamine. Our GO/KEGG analysis results indicated that its anticancer effect may be related to cell population proliferation, GTPase activity, and Apelin signaling pathway. Previous studies showed that knocking down apelin significantly suppressed cell proliferation and migration and promoted cell apoptosis in esophageal cancer cells in vitro, potentially by activating PI3K/mTOR signaling pathway [37]. GTPases were considered to play a crucial role in regulating complex cellular processes, including cell proliferation; the dysfunction of some certain GTPases that were closely related to the development and progression [38]. Taken together, our data reported here may provide mechanistic basis for the role of esketamine on the malignant proliferation of ESCC cells.

To further validate protein expression from proteomic analysis, we performed western blot and a series of bioinformatic analysis based on TCGA–ESCA database. ERCC6L, AHR and KIF2C downregulation were found to be potential mechanism of anti-tumor property of esketamine. Notably, ERCC6L was considered to be a poor prognosis marker that promotes cancer cell proliferation, migration and/or invasion, with aberrantly high expression in a wide variety of aggressive cancers, including hepatocellular cancer and non-small cell lung adenocarcinoma [27, 39]. AHR is a ligand-dependent transcription factor of the basic helix–loop–helix/Per–Arnt–Sim family. Remarkably, sustained transcriptional activation of AHR was reported to facilitate tumor development and impairs the anti-tumor immunity [30]. Thus, there are several ongoing studies targeting the AHR signaling pathway to enhance antitumor immunity and re-sensitize tumor to chemotherapy. KIF2C is a member of the kinesin-13 family that play an important role in depolymerization processes of microtubules by disassembling tubulin subunits at the filament end. Aberrant expression of KIF2C are frequently implicated in an oncogenic state and promotion of tumorigenesis in several cancer tissues, including breast cancer and hepatocellular carcinoma [28, 29, 40]. Overall, the functional analysis indicated that esketamine suppressed ERCC6L, AHR and KIF2C may be the underlying mechanisms for its anti-cancer effects, although further study is needed.

There are several limitations in our work. First, all experiments were performed in cultured ESCC cells. Further studies of animal and clinical data were required to further validate the benefits of esketamine on ESCC patients. Second, the causal relationship between the anti-tumor effects of esketamine and molecular changes found in this study warrants further study. Third, the lack of non-cancerous cell lines was used for comparison. One does not know whether its effects are unique for cancer only or not. Finally, the concentrations of esketamine used in this study were far beyond the clinical conditions. Although the previous in vitro study used the similar concentrations of S-ketamine the pancreatic cancer cells [17], caution is taken for interpreting the findings reported in the current study.

## Conclusion

**Fig. 8:**
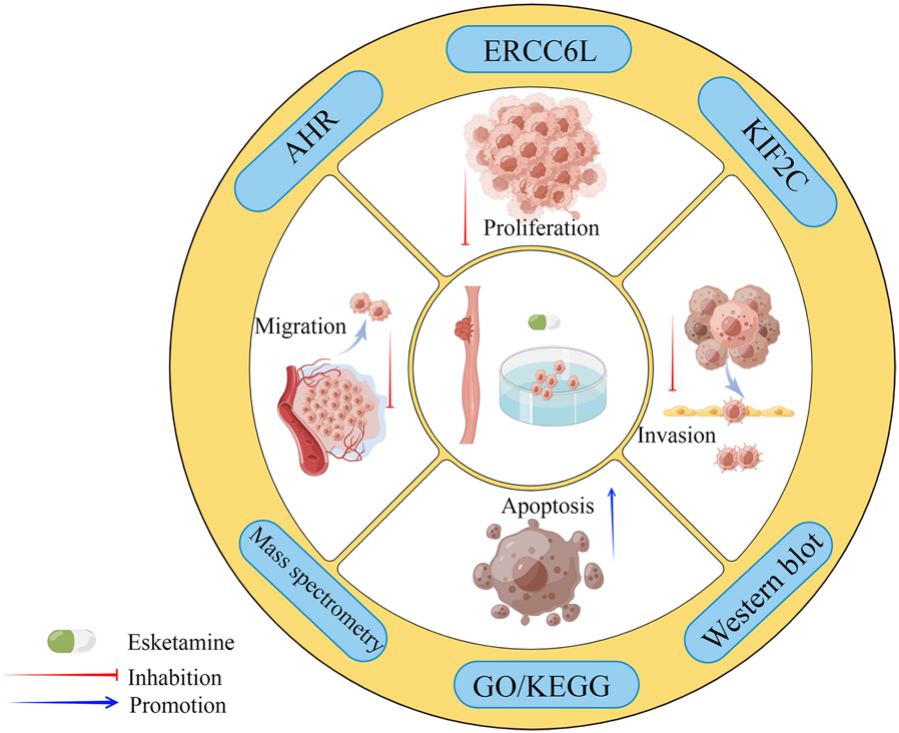
Schematic diagram depicts the antitumor effect of esketamine on esophageal squamous carcinoma cells (ESCC) in vitro and its potential molecular mechanism based on mass spectrometry. Esketamine treatment promotes apoptosis and inhibits proliferation, migration and invasion of ESCC cells, which may be related to its functional suppression of ERRCC6L, AHR and KIF2C

In summary, the present study found the tumor-suppressive effects of esketamine on ESCC cells in vitro (Fig. 8). However, its translational value is subjected to further study in in vivo and clinical settings.

### Supplementary Information

**Additional file 1:** Table S1.

## Data Availability

Data are available from the authors upon reasonable request.

## Abbreviations

AHR: Aryl hydrocarbon receptor
DEPs: Differentially expressed proteins
ESCC: Esophageal squamous cell carcinoma
ERCC6L: Excision repair cross-complementation group 6 like
GO: Gene ontologies
KEGG: Kyoto Encyclopedia of Genes and Genomes
KIF2C: Kinesin-13 family member 2C
NMDA: *N*-methyl-D-aspartate
TCGA: The Cancer Genome Atlas
