# Supplementary material for: Potential ‘anti-cancer’ effects of esketamine on proliferation, apoptosis, migration and invasion in esophageal squamous carcinoma cells
Source: Eur J Med Res. 2023 Nov 15;28:517. doi: 10.1186/s40001-023-01511-x (PMC10647146; doi:10.1186/s40001-023-01511-x)
Supplement: Supplementary file 1 — Additional file 1: Table S1. [file 40001_2023_1511_MOESM1_ESM.docx]

Table S1: 32 common downregulated DEPs, which were also highly expressed in ESCC tissue from GEPIA database.

|  |  | 1 mM esketamine vs control | | | 2 mM esketamine vs control | | |
| --- | --- | --- | --- | --- | --- | --- | --- |
| protein accession | name | Fold Change | log_2_FC | p-value | Fold Change | log_2_FC | p-value |
| Q9NVI1 | FANCI | 0.209252 | -2.256684321 | 0.02063394 | 0.06609461 | -3.91932 | 5.72E-08 |
| P35869 | AHR | 0.15831 | -2.659172572 | 0.000258894 | 0.14267486 | -2.8092 | 0.001461 |
| Q2NKX8 | ERCC6L | 0.137279 | -2.86482078 | 0.002073248 | 0.076723807 | -3.704181876 | 7.62E-05 |
| P11717 | IGF2R | 0.292945 | -1.771299684 | 0.017890383 | 0.142791882 | -2.80801 | 6.26E-07 |
| Q9BYG3 | MKI67 | 0.234233 | -2.093985664 | 0.001463594 | 0.176283391 | -2.50403 | 0.000262 |
| Q9UIG0 | BAZ1B | 0.392306 | -1.349950317 | 0.03482942 | 0.210615975 | -2.24731 | 1.52E-07 |
| Q9H9Y6 | POLR1B | 0.312237 | -1.679286908 | 0.016392564 | 0.219837968 | -2.18549 | 0.004394 |
| O95235 | KIF20A | 0.350522 | -1.512422501 | 0.036138867 | 0.225963648 | -2.14584 | 0.01727 |
| O75717 | WDHD1 | 0.380632 | -1.393531793 | 0.01801241 | 0.231631443 | -2.1101 | 3.11E-06 |
| Q00534 | CDK6 | 0.408855 | -1.290339382 | 0.006299382 | 0.314302163 | -1.66978 | 0.000221 |
| Q06481 | APLP2 | 0.249893 | -2.000616984 | 1.60E-06 | 0.32439213 | -1.62419 | 0.025003 |
| Q99661 | KIF2C | 0.482715 | -1.050756426 | 0.027191841 | 0.329420107 | -1.602 | 4.44E-06 |
| P61962 | DCAF7 | 0.482108 | -1.052572692 | 0.009595602 | 0.387808134 | -1.36659 | 0.000355 |
| Q92922 | SMARCC1 | 0.520089 | -0.943168502 | 0.023232195 | 0.388602792 | -1.36363 | 3.83E-05 |
| Q96PZ0 | PUS7 | 0.366098 | -1.449696669 | 2.59E-05 | 0.417025989 | -1.26179 | 0.025447 |
| O43823 | NCAPD2 | 0.557314 | -0.843436744 | 0.022960154 | 0.444781444 | -1.16883 | 0.000234 |
| Q9BSC4 | NOL10 | 0.540371 | -0.887977938 | 0.014465529 | 0.453355129 | -1.14129 | 0.011419 |
| Q92530 | PSMF1 | 0.55358 | -0.85313547 | 0.016019912 | 0.457034194 | -1.12963 | 0.000489 |
| O60885 | BRD4 | 0.573892 | -0.801149852 | 0.024790025 | 0.464088879 | -1.10753 | 0.000316 |
| P38571 | LIPA | 0.360591 | -1.471565069 | 0.014998031 | 0.470208645 | -1.08863 | 0.014839 |
| Q15043 | SLC39A14 | 0.589356 | -0.762788511 | 0.024684795 | 0.488772906 | -1.03276 | 0.000607 |
| Q8IY67 | RAVER1 | 0.50079 | -0.997722601 | 0.000935754 | 0.508227455 | -0.97645 | 0.019402 |
| P07996 | THBS1 | 0.511248 | -0.967905627 | 0.006015673 | 0.515368645 | -0.95632 | 0.019299 |
| P50748 | KNTC1 | 0.590406 | -0.760220567 | 0.013232548 | 0.535005862 | -0.90237 | 0.005138 |
| Q9UIG0 | SMARCA5 | 0.650871 | -0.61955686 | 0.045098733 | 0.550493417 | -0.8612 | 0.001173 |
| O95347 | SMC2 | 0.571326 | -0.807615158 | 0.005614778 | 0.55402029 | -0.85199 | 0.015627 |
| Q99519 | NEU1 | 0.64736 | -0.627360966 | 0.035988638 | 0.57189058 | -0.80619 | 0.004229 |
| P11388 | TOP2A | 0.59778 | -0.742312493 | 0.009807616 | 0.576098598 | -0.79561 | 0.017899 |
| Q9ULX6 | PRPF40A | 0.553426 | -0.853538321 | 0.002250375 | 0.580630288 | -0.78431 | 0.045015 |
| P54687 | BCAT1 | 0.602719 | -0.73044239 | 0.010809434 | 0.58315262 | -0.77805 | 0.021144 |
| Q9NR30 | DDX21 | 0.607874 | -0.718156737 | 0.009178017 | 0.611607663 | -0.70932 | 0.036783 |
| Q8N3U4 | STAG2 | 0.611565 | -0.709422217 | 0.009277775 | 0.624171203 | -0.67999 | 0.047191 |
